# Tailoring Practically Accessible Polymer/Inorganic Composite Electrolytes for All-Solid-State Lithium Metal Batteries: A Review

**DOI:** 10.1007/s40820-022-00996-1

**Published:** 2023-01-31

**Authors:** Hongmei Liang, Li Wang, Aiping Wang, Youzhi Song, Yanzhou Wu, Yang Yang, Xiangming He

**Affiliations:** Institute of Nuclear and New Energy Technology, Tsinghua University, Beijing, 100084 People’s Republic of China

**Keywords:** Polymer, Inorganic composite electrolytes, All-solid-state lithium metal batteries, Fillers, Ionic conductivity, High voltage

## Abstract

**Highlights:**

- The current issues and recent advances in polymer/inorganic composite electrolytes are reviewed.
- The molecular interaction between different components in the composite environment is highlighted for designing high-performance polymer/inorganic composite electrolytes.
- Inorganic filler properties that affect polymer/inorganic composite electrolyte performance are pointed out.
- Future research directions for polymer/inorganic composite electrolytes compatible with high-voltage lithium metal batteries are outlined.

**Abstract:**

Solid-state electrolytes (SSEs) are widely considered the essential components for upcoming rechargeable lithium-ion batteries owing to the potential for great safety and energy density. Among them, polymer solid-state electrolytes (PSEs) are competitive candidates for replacing commercial liquid electrolytes due to their flexibility, shape versatility and easy machinability. Despite the rapid development of PSEs, their practical application still faces obstacles including poor ionic conductivity, narrow electrochemical stable window and inferior mechanical strength. Polymer/inorganic composite electrolytes (PIEs) formed by adding ceramic fillers in PSEs merge the benefits of PSEs and inorganic solid-state electrolytes (ISEs), exhibiting appreciable comprehensive properties due to the abundant interfaces with unique characteristics. Some PIEs are highly compatible with high-voltage cathode and lithium metal anode, which offer desirable access to obtaining lithium metal batteries with high energy density. This review elucidates the current issues and recent advances in PIEs. The performance of PIEs was remarkably influenced by the characteristics of the fillers including type, content, morphology, arrangement and surface groups. We focus on the molecular interaction between different components in the composite environment for designing high-performance PIEs. Finally, the obstacles and opportunities for creating high-performance PIEs are outlined. This review aims to provide some theoretical guidance and direction for the development of PIEs.
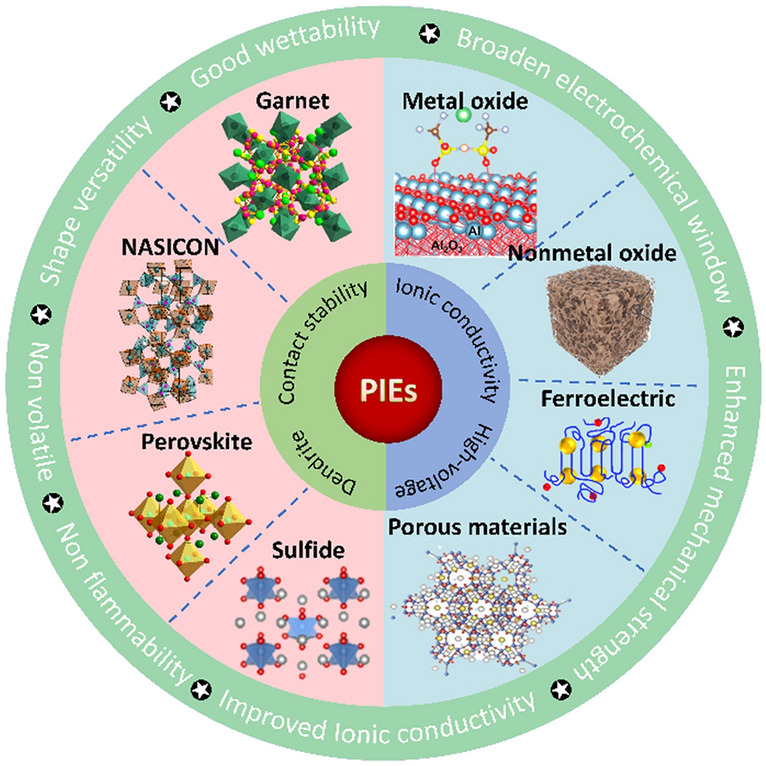

## Introduction

The ever-increasing energy consumption sparks widespread interest in energy-efficient storage and flexible conversion. Lithium-ion batteries (LIBs) have been heavily marketed in consumer electronics and traffic electrification owing to their eco-friendliness, high energy density and working voltage [1–3]. Currently, the energy density of LIBs has approached 260 Wh kg^−1^ and is challenging to break through [4, 5]. Meanwhile, LIBs have repeatedly experienced catastrophic failure in recent years, resulting in severe property damage and raising public concern. Developing LIBs with high energy density and safety has become unremitting pursuit. Organic liquid electrolyte frequently employed in commercial LIBs is blamed for thermal runaway [6]. It has volatility and flammability, posing safety issues about leakage and fire. The constituent solvents such as ethylene carbonate have strong reactivity with lithium metal anodes (LMAs) known as “holy grail” anodes, causing dendrite growth and continual side reactions [7–9]. Solid-state electrolytes (SSEs) can effectively enhance safety by eliminating the flammable liquid electrolyte. They can inhibit the dissolution of transition metal ions of the cathode materials and block the by-product cross talk between the electrodes [10]. SSEs can also limit the shuttle effect of polysulfide in lithium–sulfur batteries and reduce the cross talk of O_2_ and H_2_O as well as the nucleophilic attack of reduced oxygen in lithium-oxygen batteries [11, 12]. Some SSEs exhibit thermodynamic/electrochemical compatibility on the interfaces of LMAs, which further broaden the electrochemical window and enhance the energy density [13].

**Fig. 1:**
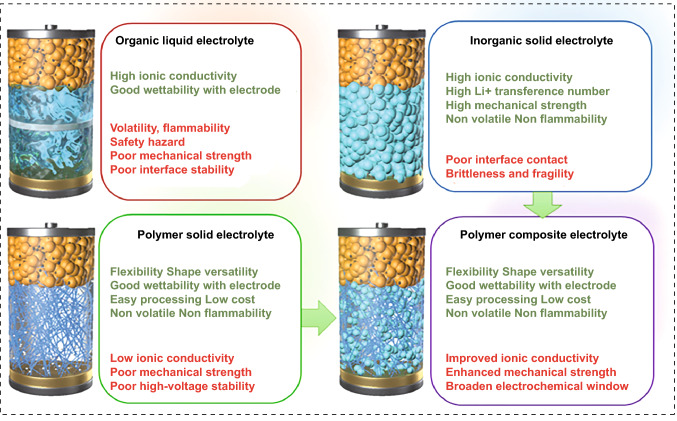
Performance comparison of different electrolytes

SSEs can be categorized into two groups: polymer solid-state electrolytes (PSEs) and inorganic solid-state electrolytes (ISEs). Single PSEs and ISEs are challenging to fulfill general requirements, such as adequate ionic conductivity (> 10^–4^ S cm^−1^), high operating voltage (up to 4–5 V vs. Li/Li^+^), appropriate mechanical strength (> 6 GPa) and excellent interfacial contact (Fig. 1) [14, 15]. PSEs exhibit good elasticity and adaptability to volume variations, which are widely used for flexible batteries. However, the polymers crystallize easily at ambient temperature, resulting in limited ionic conductivity [16]. The thermodynamic instability of the interface restricts their compatibility with high-voltage cathode materials and the inferior mechanical properties cannot suppress dendrite growth [17, 18]. ISEs own acceptable ionic conductivity, extensive electrochemical window and satisfactory mechanical strength, while their brittleness and fragility cause poor machinability and large contact resistance. Recently, researchers have been committed to integrating inorganic fillers into PSEs to form polymer/inorganic composite electrolytes (PIEs) and realize the synergistic effect of different materials. Inorganic fillers not only increase the mechanical strength of the polymer matrix but also act as plasticizers, preventing polymer crystallization and boosting the ionic conductivity of the electrolyte [19–21]. The interaction of the fillers with the polymer increases the redox stability of the electrolyte, hence extending the electrochemical window [22]. PIEs with sufficient ionic conductivity, electrochemical stability and outstanding mechanical strength represent tremendous potential for the next generation of LIBs.

Numerous inorganic fillers have emerged to enhance the performance of PIEs, including metal oxides, ceramic Li^+^ conductors and novel porous materials like metal–organic frameworks (MOFs). Despite extensive researches asserting that certain fillers have the potential to dramatically enhance PIE performance, the mechanism underlying these improvements lacks in-depth understanding and sortation. This review provides a comprehensive summary of the existing challenges and current advancements in PIEs. The properties of the PIEs are profoundly influenced by the nature of ceramic fillers including content, morphology, arrangement and surface groups. The molecular interaction in different phases and interface regions are highlighted to understand the improvement. The major purpose of this review is to propose alternative solutions to overcome the defects of PIEs and inspire the engaged contributors and new entrants to explore scalable strategies for the industrialization of PIEs.

## Key Issues in the Development of PIEs

**Fig. 2:**
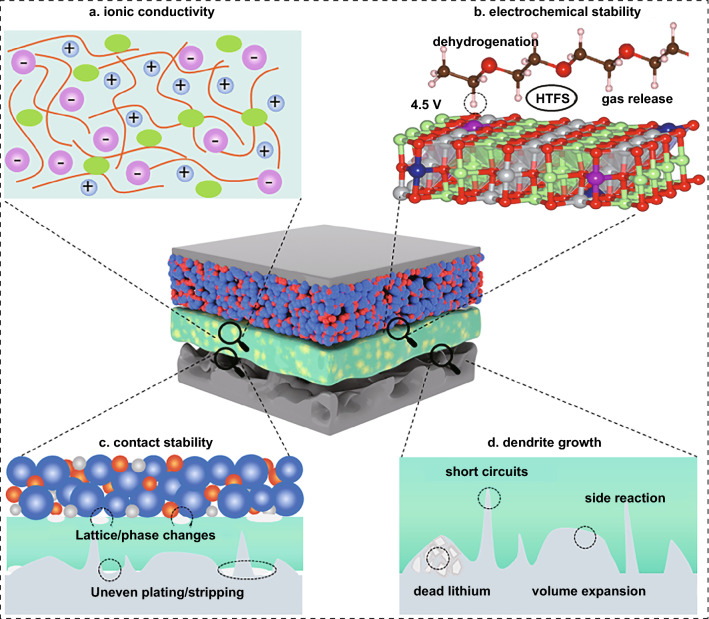
Key issues in the development of PIEs

The key issues in the development of PIEs are illustrated in Fig. 2. PIEs must be engineered to be thin (thickness < 30 μm) and have fast Li^+^ transport capability to compete with the available commercial liquid LIBs [23]. Besides, PIEs should match the electrodes with high loading, specific capacity and working voltage to gain advantages in energy density and power capability. Combining SSEs with 4 V-class cathode and LMAs can increase the energy density, which also poses a significant challenge since it may cause performance deterioration due to high reactivity between electrolyte and charged electrodes [24]. The poor point-to-point contact generates a substantial interface resistance and uneven distribution of local current density, driving dendritic growth. Periodic volume changes of the electrode lead to the formation and accumulation of structural stress, which will deteriorate the ion transport on the electrode/electrolyte interface [25]. Improving the voltage window and developing a stable interface of PIEs are crucial challenges for achieving high-performance all-solid-state batteries (ASSBs).

### Lithium-Ionic Conductivity

**Fig. 3:**
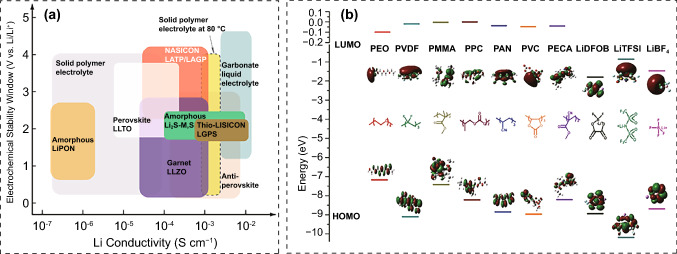
**a** Ionic conductivity and electrochemical window of different SSEs [33]. Copyright 2020, American Chemical Society. **b** HOMO and LUMO values of different polymers and lithium salts [32]. Copyright 2019, John Wiley and Sons Publisher

Ionic conductivity is a critical metric for accessing the migratory ability of Li^+^ in electrolytes. It is proportional to carrier concentration and transference number (t_+_). The ionic conductivity of commercial liquid organic electrolytes can approach 10^–3^–10^–2^ S cm^−1^, while the ionic conductivity of PSEs is less than 10^–4^ S cm^−1^ at room temperature [26]. PSEs with conductivity less than 5 × 10^–4^ S cm^−1^ are incapable of meeting the operational requirements of thick electrodes (thickness > 70 μm) [27], and gain no advantage in terms of energy density. According to the free-volume model, polymer matrix transfers Li^+^ ions through the polar sites and local segmental motions in amorphous regions [28, 29]. The ion diffusion kinetics in crystalline region is negligible. Reducing the crystallinity of polymer matrix at normal temperature is a crucial method for enhancing conductivity. However, lowering crystallinity reduces polymer strength, causing the polymer behaves as a viscous liquid and incapable of forming a self-supporting membrane. The balance between conductivity and mechanical strength raises concerns regarding polymers as hosts. Given that the reported ionic conductivity of certain ISEs has reached 10^–3^ S cm^−1^ and they feature great mechanical strength, the development of polymer/ceramic composite electrolytes should be a viable solution to enhance ion conductivity to some extent (Fig. 3a) [30, 31]. Ionic conductivity of PIEs is primarily influenced by interactions between Li^+^, anions, polymers and fillers [32]. The ion–dipole interaction between ions and the polymer matrix impacts the concentration of free Li^+^. The Lewis acid–base interaction generated by inorganic fillers influences polymer segment motion, lithium salt solubility and Li^+^ ion diffusion behavior. Making full use of the interaction between different components to optimize ionic conductivity has emerged as a primary focus for developing PIE.

The t_+_ quantifies the contribution of Li^+^ to the transport charge. Since anions do not engage in reversible electrochemical reactions, their migration cannot transfer effective charges [34]. Nevertheless, for anions with a large volume and mass, their transfer number is always bigger than that of Li^+^; hence, the t_+_ of PSEs is always less than 0.5 [35]. t_+_ is determined by the ability of polymer to dissociate lithium salt, and thus polymer with a high dielectric constant and lithium salt with a low lattice energy can achieve high t_+_ [36, 37]. Besides, the transport of Li^+^ strongly depends on the segmental movement of the amorphous region in the polymer matrix. Polymer with a low glass transition temperature (*T*_g_) can facilitate the segment movement and enhance t_+_. Adding fillers in polymer can change the local environment of Li^+^ ions. Especially, the strong interaction between fillers and anions results in the dissociation of lithium salts and an increase in t_+_. Fixing anions with fillers to increase t_+_ can reduce the concentration polarization on the electrode and inhibits fractal dendrites caused by the depletion of Li^+^ on the anode.

### Electrochemical Stability

The electrolyte decomposes when the working potential of the battery exceeds its redox potential window [38, 39]. To achieve high-voltage stability, PIEs require every component has a HOMO energy level less than the Fermi energy of cathode. The HOMO values of most polymers are greater than those of lithium salts, indicating that the polymers preferentially undergo interfacial side reactions (Fig. 3b) [32]. Furthermore, adding lithium salt reduces the oxidation stability of polymers because the anions shield positive charges on the chains [40]. Electrolytes based on PEO are typically utilized for 3 V-grade cathode materials due to the labile lone pairs on the ether-oxygen atoms in the PEO chains [41]. Yu et al. found that the C-H bonds became weak after partially oxidizing the ether-oxygen atoms, causing the H protons to be carried away by the TFSI^−^ and generate hydrogenated HTFSI. As a potent acid, it can impair interface and produce H_2_ on the anode [42]. In addition, the cathodes such as LiNiO_2_, LiCoO_2_ and LiNi_x_Co_y_Mn_1−*x*−*y*_O_2_ possess large specific surface areas and show strong catalytic ability, due to the transition metal ions or conductive carbon, hence accelerating electrolyte degradation [43, 44]. The molecule interaction of the components changes the chemical environment of the polymers, which consequently affects their HOMO value. Incorporating inorganic fillers can improve the oxidative stability of polymers via Lewis acid–base interaction, hydrogen-bonding or dipolar interactions between the lone pairs of polymers and the surface groups of fillers [32, 45]. Cui et al. reported that the ether-oxygen segments in the polymer matrix can interact with the P atoms in the Li_6_PS_5_Cl fillers, thereby reducing the HOMO energy level of the polymer and widening the electrochemical window [46]. Chen et al. found that the strong Lewis acid–base interaction between anions and the surface groups of Li_7_La_3_Zr_2_O_12_ fillers can decrease the oxidation of anions [47]. Meanwhile, combining anions with the fillers can diminish the shielding effect of anions on the positive charges of polymers and effectively stabilize the polymers at high voltage. Furthermore, rational design of polymer and inorganic Li^+^ conductors can inhibit the direct contact of unstable interface and improve the compatibility with Li and high-voltage cathode [48]. Specifically, most polymers are stable at the Li anode but poor at the high-voltage cathode, whereas certain inorganic oxides and sulfides are the exact reverse. Properly designing PIEs with two or more layers of vertical heterostructure provides a viable option for concurrently meeting cathode and anode requirements, exploring a new pathway for high-voltage ASSBs.

### Dendrite Inhibition

**Fig. 4:**
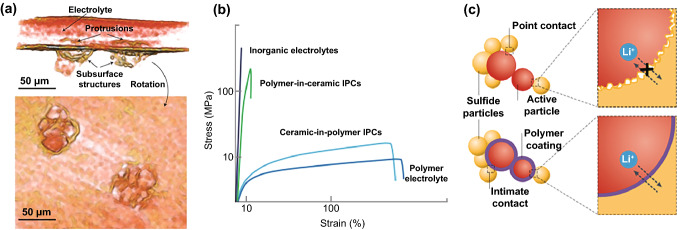
**a** 3D reconstructed volumes of the dendrite located on the subsurface below the polymer/electrode interface [54]. Copyright 2014, Springer Nature. **b** Stress–strain curves of different SSEs [23]. Copyright 2021, Springer Nature. **c** PIEs can improve the contact stability with electrode [23]. Copyright 2021, Springer Nature

LMA possesses unique superiority in energy density because it owns the lowest molar mass and reduction potential among metallic elements [49, 50]. However, notorious dendrite propagation gives rise to large volume expansion, low reversibility and potential safety hazards [51]. In polymer electrolytes, heterogeneous interface, limited ion transport and low mechanical strength are the primary reasons driving dendrite growth [52]. Firstly, solid electrolyte interface (SEI) realizes the dynamic passivation of the electrode, which expands the electrochemical window of LMBs to a certain extent [53]. However, the heterogeneous SEI induces uneven Li^+^ flux on the anode, triggering the propagation of mossy and whiskery dendrites (Fig. 4a) [7, 54]. Isotropic inert interface with uniform ionic conductivity can effectively homogenize lithium flux; thus, dendrite growth can be effectively alleviated by constructing a stable electrolyte layer on the anode. Furthermore, the limited transport results in local ion depletion on the interface, creating a space charge layer (SCL) [55]. The large electric field in the SCL leads to electric convection and rapid growth of fractal dendrites. Fixing anions to enhance t_+_ and prevent SCL formation is regarded as an effective method for inhibiting dendrites [56]. Additionally, enhancing mechanical strength can regulate Li nucleation and growth by altering the surface energy at the Li top surface [57]. According to the theoretical model proposed by Monroe and Newman, lithium dendrites can be eliminated when the surface shear modulus is at least 2–3 times that of metallic lithium (4.5 GPa) [58]. The polymer electrolytes have a low shear modulus (typically < 0.1 GPa) and are incapable of inhibiting Li dendrites (Fig. 4b). Viswanathan et al. further established a universal criterion for stable electroplating using the shear modulus ratio of SSEs and lithium anode ($G_{{{\text{SSE}}}}$/$G_{{{\text{Li}}}}$) and the molar volume ratio of Li^+^ ions and lithium anode ($V_{{{\text{Li}}^{ + } }} /V_{{{\text{Li}}}}$) [59]. They concluded stable electroplating necessitated the use of SSEs with a high (low) Li molar volume and high (low) shear modulus. PSEs have soft texture and low shear modulus, while the formation of Li^+^ solvated “cages” leads to high volume expansion and high $V_{{{\text{Li}}^{ + } }}$, causing they cannot inhibit dendrites. To verify the feasibility of this criterion, Helms et al. prepared nano-LiF@polymer PIEs by in situ cation metathesis [60]. The modified PIEs had minimally reconfigurable, ceramic-like, ion-conducting domains contained in a soft, polymer-like matrix with a low shear modulus, which can inhibit the growth of dendrites.

### Contact Stability

During reciprocating charge and discharge, the electrode materials may undergo lattice and phase changes, causing volume fluctuation and particle pulverization [61, 62]. Inadequate contact between the electrode and PIEs leads to high contact resistance even complete loss of electric disconnection (Fig. 4c). Besides, the uneven plating/stripping behavior of metallic lithium reduces the effective contact area, hence exacerbating the inhomogeneous distribution of Li^+^ flux. A decent electrolyte design requires a compromise between the appropriate modulus and surface adhesion [63].

In addition to the typical issues listed above, PIEs face additional challenges with some specific energy storage systems. Lithium–sulfur batteries have an overwhelming advantage in energy density (500–600 Wh kg^−1^), which partly attributes to the reduction of S to Li_2_S yields a high specific capacity of 1675 mAh g^−1^. However, the shuttle effect of soluble polysulfide intermediates (Li_2_S_n_, 3 ≤ n ≤ 8) results in the rapid attenuation of capacity and low coulombic efficiency [64]. Some polymers such as PEO have a strong solvation effect on polysulfides at high temperatures, causing polysulfides to dissolve in polymers and trigger side reactions [65]. The polysulfides on the anode induce uneven plating/stripping behavior of lithium and further deterioration of interface contact [66]. As physical barriers, inorganic fillers can reduce the contact with polymers [67]. Meanwhile, they can adsorb polysulfides and mitigate the shuttle effect by forming chemical bonds with polysulfides [68]. Li–O_2_ (air) batteries also have a much higher energy density (~ 950 Wh kg^−1^) than the existing graphite||layered ternary cathode system. Polymers with non-toxic, non-combustible and nonvolatile characteristics provide feasible solutions to solve safety problems. However, most routinely used polymers, including PAN, PVDF, PVDF-HFP and PEO, are reactive to reduction products such as Li_2_O_2_ [69, 70]. Fortunately, Lewis acid base interaction between inorganic fillers and polymers can improve the electrochemical stability of the polymers [71, 72]. The charge transfer of Li–O_2_ batteries using liquid electrolyte occurs at the solid–liquid-gas interface, while that of Li–O_2_ batteries using PIEs occurs at the solid–gas interface. Due to the increased contact resistance, the reaction rate would be drastically slowed down. It is essential to develop catalysts to accelerate the kinetics of conversion reaction.

## Fillers of PIEs

### Components of PIEs

**Fig. 5:**
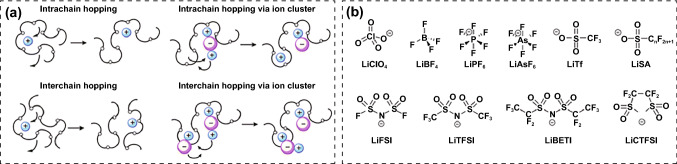
**a** Li^+^ ions diffuse through the polar groups and segment movement of the polymer chains [26]. Copyright 2018, Royal Society of Chemistry. **b** Common lithium salts used in PIEs [26]. Copyright 2018, Royal Society of Chemistry

PIEs are made up of polymer matrix, lithium salt and ceramic filler. Wright et al. proposed that alkali metal salts mixed with polyethylene oxide (PEO) could conduct ions in 1973 [73]. And then Armand used the composite as electrolyte in batteries [74]. Subsequently, a broad array of polymers, including polyvinylidene fluoride (PVDF), polymethyl methacrylate (PMMA), polyacrylonitrile (PAN) and poly(vinylidene fluoride-co-hexafluoropropylene) (PVDF-HFP), emerged as matrices [73, 75]. It is challenging for a single polymer to satisfy all the requirements as electrolyte material (Table 1). By combining the benefits of several hosts, polymer/polymer cooperation offers the chance to create superior polymer matrices. Copolymerization, cross-linking, interpenetration and blending are the most explored techniques in this field [22]. These polymer segments typically include polar groups to dissolve lithium salts and transfer Li^+^ ions, such as C=O, –O–, –N–, C=N and –P– [37]. Li^+^ ions coordinate with polar groups on the polymer chains at certain places and generate free volume by local segment movement of the polymer chains, allowing Li^+^ to be transmitted within and between the chains [26] (Fig. 5a). Table 1 lists the fundamental characteristics of typical polymer matrices in terms of *T*_g_ and melting point (*T*_m_). These two parameters govern the conductivity of Li^+^ ions. Specifically, T_g_ is crucial for the phase transition of polymer electrolytes since most studies hold that Li^+^ ion transport only takes place in the amorphous zone above *T*_g_.

**Table 1:** Main properties of polymer matrix [76–79]

Polymer	Repeating unit	*T*_g_ (°C)	*T*_m_ (°C)	Advantages	Disadvantages
PEO	[CH_2_CH_2_O]n	− 64	65	Strong electron donating ability, soft molecular chain, good thermal stability	Crystallization occurs at low temperature, low ionic conductivity and t_+_
PVDF	[CH_2_CF_2_]n	40	171	High melting point, good thermal stability and electrochemical stability promote the ionization of lithium salts	High crystallinity, low ionic conductivity
PMMA	[CH_2_C(CH_3_)COOCH_3_]n	5	Amorphous	Stable to metal lithium and the passivation film has a small impedance	Poor film-forming ability, flexibility and mechanical strength
PAN	[CH_2_CH(CN)]n	25	317	Good thermal stability, flame retardancy, good mechanical properties and ionic conductivity	C=N group can react with LMAs

Ordinary lithium salts usually contain the characteristics of large anionic radius and delocalization charge, such as LiPF_6_, LiFSI, LiTFSI and LiClO_4_, which have high solubility in polymers and easily generate stable SEI [26, 80] (Fig. 5b). Ceramic fillers can be classified as either inert or active fillers depending on whether they can conduct Li^+^ ions. The inert fillers include SiO_2_, ZrO_2_, Al_2_O_3_, Y_2_O_3_, LiAlO_2_, and the active fillers include garnet, NASICON, perovskite, sulfide, Li_3_N, etc. [71, 81–84]. Both inert and active fillers can be utilized as plasticizers to diminish the crystallization, hence facilitating the movement of Li^+^ ions. As fast ion conductors, active fillers can also promote Li^+^ diffuse through the defects or vacancies in the crystal structure, such as Schottky defects and Flenker defects, thus enhancing the ionic conductivity. If the active fillers are highly concentrated, Li^+^ ions can diffuse through the permeation network provided by continuous filler particles [85]. In this case, the polymer matrix only acts as a flexible host and is not responsible for Li^+^ ion diffusion. Therefore, high ion conductivity and t_+_ can also be achieved without lithium salts [86–88].

### Inert Fillers

The thermal and mechanical strength of the polymer matrix can be improved by inert fillers. Moreover, fillers dispersed in the polymer matrix typically have tiny particle sizes and large specific surface areas, creating abundant interface with massive defects and high reactivity, which easily interact with other components [89]. Interaction between components affects the ionic conductivity and electrochemical stable window (ESW). Precisely regulating the intermolecular force is essential for achieving PIEs with high performance [89].

**Fig. 6:**
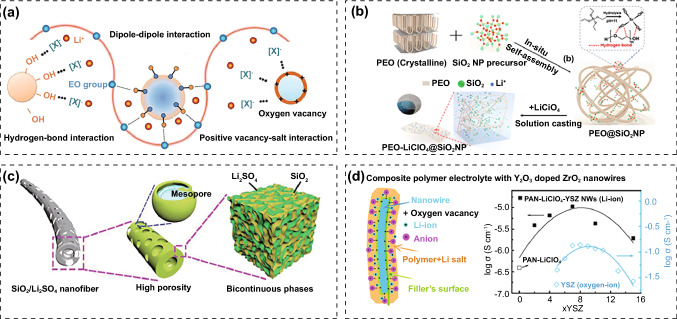
**a** Lewis acid–base interaction between different components in PIEs [32]. Copyright 2019, John Wiley and Sons Publisher. **b** Synthetic process of the PEO-LiClO_4_@SiO_2_ PIEs [94]. Copyright 2020, American Chemical Society. **c** Schematics of the PEO-LiTFSI@Li_2_SO_4_ modified SiO_2_ PIEs [95]. Copyright 2019, John Wiley and Sons. **d** Structure and ionic conductivity of PAN-LiClO_4_@Y_2_O_3_-doped ZrO_2_ PIEs [96]. Copyright 2016, American Chemical Society

Inert fillers can weaken the interaction among the chains and increase free volume in the polymer matrix, which speeds up segmentation dynamics and delays polymer crystallization. Furthermore, fillers with Lewis acidic surface can interact with the anions [90]. As a result, the newly established hydrogen bonds make the fillers become cross-linking centers between polymer and anions, further disrupting the crystallinity (Fig. 6a) [32, 91, 92]. Instead, the fillers with Lewis basic surface can interact with Li^+^, causing the decrease of t_+_. Neutral fillers interact weakly with lithium salts and polymer, hence having a negligible effect on the transport characteristics. Therefore, fillers with Lewis acidic surface are more favorable to Li^+^ ion diffusion. The inert fillers can also facilitate salt dissociation and increase Li^+^ ion concentration. Fixing anions on fillers can prevent anion–polymer interaction to increase the oxidation stability of PIEs [45, 93]. Meanwhile, most inorganic fillers are stable at high voltages. Well-designed PIEs can broaden ESW by inhibiting the direct contact of thermodynamically unstable components to realize compatibility with LMAs and high-voltage cathode. The recent research on PIEs with inert fillers and their properties is presented in Table 2.

**Table 2:** PIEs filled with passive fillers and their properties

Polymer matrix	Lithium salt	Fillers	Ionic conductivity (S cm^−1^)	ESW (V)	t_+_	References
PEO	LiTFSI	BaTiO_3_	1.3 × 10^–4^ (30 °C)	4	–	[97]
PEO	LiClO_4_	BaTiO_3_	1.2 × 10^–3^ (70 °C)	–	0.37	[98]
PEO	LiCF_3_SO_3_	LiNbO_3_	2 × 10^–4^ (85 °C)	–	0.52	[98]
PEO	LiTFSI	SSZ-13	1.7 × 10^–2^ (60 °C)	4.65	0.84	[99]
PEO	LiTFSI	UiO-66	2.9 × 10^–4^ (60 °C)	4.3	0.52	[100]
PEO	LiTFSI	UiO-66-NH_2_	6.3 × 10^–4^ (60 °C)	4.97	0.72	[101]
PEO	LiTFSI	Al_2_O_3_	9.6 × 10^–4^ (25 °C)	5	0.81	[102]
PEO-PMMA	LiTFSI	Al_2_O_3_	9.4 × 10^–7^ (25 °C)	4.9	–	[103]
PVDF-HFP	LiPF_6_	PMMA-ZrO_2_	3.6 × 10^–3^ (25 °C)	5	0.41	[104]
PPC	LiTFSI	Lithiated TiO_2_	1.2 × 10^–4^ (25 °C)	4.6	0.58	[105]
PEO	LiTFSI	Ti^3+^-doped TiO_2_	1 × 10^–4^ (25 °C)	5.5	0.36	[106]
PEMA/PVAC	LiClO_4_	TiO_2_	2.7 × 10^–3^ (25 °C)	2.1	–	[107]
PPC	LiTFSI	TiO_2_ nanorods	1.2 × 10^–4^ (25 °C)	4.6	–	[108]
PVDF-PVC	LiBOB	TiO_2_	5.4 × 10^–4^ (25 °C)	–	–	[109]
PEO	LiBF_4_	ZrO_2_	4.4 × 10^–4^ (80 °C)	–	0.68	[110]
PVDF-HFP	LiClO_4_	ZrO_2_	2.5 × 10^–3^ (25 °C)	5	0.57	[111]
PVDF-PVC	LiBOB	ZrO_2_	1.5 × 10^–3^ (70 °C)	–	–	[112]
PEO	LiTFSI	BaTiO_3_	1.3 × 10^–4^ (30 °C)	4	–	[97]
PEO	LiTFSI	Mg_2_B_2_O_5_	1.5 × 10^–4^ (40 °C)	4.75	0.44	[113]
PEO	LiTFSI	Al_2_Si_2_O_5_(OH)_4_	1.1 × 10^–4^ (25 °C)	6.35	0.4	[114]
PEO	LiFSI	MIL-53(Al)	3.4 × 10^–3^ (120 °C)	5.1	0.34	[115]
PEO	LiTFSI	UiO-66	1.3 × 10^–4^ (30 °C)	4.5	0.35	[116]
PEO	LiTFSI	Al-BTC	~ 1 × 10^–5^ (30 °C)	> 3.8	0.55	[117]
PEO	LiTFSI	Al-TPA	1 × 10^–4^ (60 °C)	> 3	–	[118]
PAN	LiClO_4_	Hydrophobic clay	2.4 × 10^–4^ (25 °C)	4.75	0.12	[119]
PAN	LiClO_4_	TiO_2_	1.8 × 10^–4^ (25 °C)	–	–	[120]
PAN	LiClO_4_	Graphene oxide	4 × 10^–4^ (30 °C)	4.3	0.4	[121]
PMMA	LiCF_3_SO_3_	ZrO_2_-TiO_2_	1.2 × 10^–5^ (30 °C)	> 2.5	–	[122]
PMMA	LiClO_4_	MgO	7.7 × 10^–6^ (30 °C)	5.1	–	[123]
PMMA	LiTFSI	SiO_2_	2.4 × 10^–6^ (30 °C)	5.1	–	[124]
PMMA	LiTFSI	SiO_2_	7.3 × 10^–5^ (30 °C)	–	–	[125]

#### Oxide Materials

Al_2_O_3_ is inexpensive and widely available with robust thermal stability and is one of the earliest materials used as filler [126–128]. Pereira et al. reported that the addition of Al_2_O_3_ did not change the *T*_g_ of PEO-LiClO_4_, but increased amorphous regions, thereby promoting the segment mobility and the transport of Li^+^ ions [129]. Wieczorek and Chen used Al_2_O_3_ with two distinct properties as fillers to demonstrate the validity of Lewis acid–base theory in elucidating the modification of ionic conductivity [130, 131]. Fourier transform infrared spectroscopy (FTIR) showed that Al_2_O_3_ with acidic groups enhanced the interaction with ClO_4_^−^, thus promoting the dissolution of LiClO_4_. Therefore, O atoms on Al_2_O_3_ with basic groups can interact with Li^+^, which increased free anions and diminished the t_+_.

SiO_2_ is easily accessible and rich in reserves and is commonly utilized as filler material [132–134]. Zhang et al. constructed a three-dimensional network of PEO-LiClO_4_@SiO_2_ by in situ hydrolysis reaction (Fig. 6b) [94]. SiO_2_ promoted the segmental motion by the synergistic effect of Lewis acid–base and hydrogen bond. In addition, the enhanced interfacial stability allowed for an ESW of up to 4.8 V at 90 °C. Lu et al. created Li_2_SO_4_-modified SiO_2_ nanofibers through electrospinning and calcination (Fig. 6c) [95]. The doping of Li_2_SO_4_ enhanced the ionic conductivity of SiO_2_ and the wettability to the polymer. Meanwhile, the created mesopores encouraged anion absorption. After integrating PEO-LiTFSI matrix, the nanofiber networks can produce rapid and continuous Li^+^ diffusion routes. The sturdy 3D network served as a solid skeleton, reinforcing the entire membrane and inhibiting dendrite growth.

TiO_2_ has a high dielectric constant (*ε* > 180) and strong Lewis acid–base action, making it a popular choice as a filler for PIEs. Ghosh et al. explored the impact of TiO_2_ nanoparticles on the characteristics of the PMMA-LiClO_4_ [135]. 1 wt% addition of TiO_2_ raised the ionic conductivity of the PIEs to 3 × 10^–4^ S cm^−1^ at room temperature. It contributed to that the strong interaction between TiO_2_ nanoparticles and ClO_4_^−^ inhibited ion pair formation and increased free carriers. Lithium-ion poly (ethyl citrate) embedded with TiO_2_ nanoparticles was in situ produced by thermal-initiated polymerization [136]. Polymer esterification catalyzed in situ hydrolysis of titanium alkoxide, leading to the production of nano-TiO_2_. As the increase in TiO_2_ concentration, polymerization of PIEs decreased and the thermal stability improved marginally. The addition of 20 wt% TiO_2_ to the PIEs increased ionic conductivity by two orders of magnitude (1.74 × 10^–4^ S cm^−1^).

ZrO_2_ has good chemical and thermal stability. In addition, ZrO_2_ nanoparticles have Lewis acidity, which can attract anions and encourage lithium salt dissociation [137]. Jing et al. fabricated polypropylene oxide (PPO)-based PIEs by combining the bis[3-(methyldimethoxysilyl)]-terminated PPO (BSPPO) oligomers with ZrO_2_ nanofillers, succinonitrile (SN) plasticizer and cellulose membrane (CM) framework. LiBOB was used to trigger the cross-linking of BSPPO oligomers. ZrO_2_ nanofillers decreased the *T*_g_ of the polymer and promoted the dissociation of LiTFSI. The ionic conductivity was further increased by the SN, which was an efficient ionizer. The prepared PPO-LiTFSI@ ZrO_2_ had good flexibility, high ionic conductivity (9.62 × 10^–4^ S cm^−1^), excellent thermal and electrochemical stability (5 V) [138]. Cui et al. employed Y_2_O_3_-doped ZrO_2_ to tailor the PAN-LiClO_4_ (Fig. 6d). High concentration of oxygen vacancies in ZrO_2_ can be created by doping with Y^3+^ with a low oxidation state. The positively charged oxygen vacancies as Lewis acid sites can combine with ClO_4_^−^ to liberate additional Li^+^ ions, which increased the conductivity to 1.07 × 10^–5^ S cm^−1^ and the t_+_ rose to 0.56 [96].

#### Ferroelectric Materials

Ferroelectric materials with permanent dipoles have strong Lewis acid–base characteristics, which are also employed as fillers [97, 139]. Due to their unique crystal structure, ferroelectric materials exhibit spontaneous polarization. They can effectively alleviate the generation of SCL formed by Li^+^ depletion due to the large chemical potential difference at the electrode/electrolyte interface [140, 141]. Sohn et al. incorporated BaTiO_3_, PbTiO_3_ and LiNbO_3_ into the PEO polymer [98]. The fillers decreased the contact resistance between the LMAs and the electrolyte, increasing the mechanical strength and Li^+^ ionic conductivity.

#### Porous Materials

Porous materials, such as zeolite and MOFs, provide an adaptable pore structure and large specific surface area, hence generating abundant contact sites [142]. In addition, the channel structure has a nanoscale effect that allows for the effective regulation of charged particle adsorption. Moreover, these materials possess excellent thermal stability and mechanical properties which have been extensively explored as inorganic fillers.

**Fig. 7:**
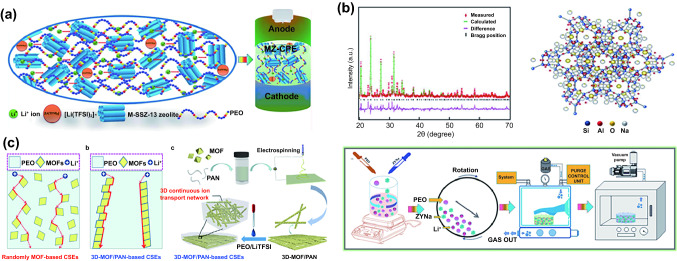
**a** Schematic of the PIEs with M-SSZ-13 zeolite as the filler [143]. Copyright 2021, Royal Society of Chemistry. **b** Structure of the YNa zeolite and schematic of the synthesis process of PEO-LiFSI@YNa [99]. Copyright 2021, Royal Society of Chemistry. **c** Synthetic process and ion transport channels of 3D PAN/PEO-LiTFSI@UIO-66 PIEs [100]. Copyright 2022, Elsevier

Zeolites are widely available from nature and possess ultra-high structural stability. Kim et al. treated the surface of aluminosilicate zeolite (SSZ-13) with polyacrylic acid (Fig. 7a) [143]. SSZ-13 with a hydrophobic surface enhanced the dispersion of LiTFSI in PEO and provided continuous channels for Li^+^ diffusion. It increased dissociation of LiTFSI and liberation of Li^+^ ions. The conductivity of PEO-LiTFSI@SSZ-13 was increased to 5.34 × 10^–2^ cm S^−1^ (70 °C) with a t_+_ of 0.85. The ASSBs assembled with Li and LiFePO_4_ delivered capacity retention of 94.1% after 80 cycles at 60 °C. Additionally, they employed YNa zeolite as a ceramic filler and combined it with PEO-LiFSI to create PIEs (PEO-LiFSI@YNa) (Fig. 7b) [99]. The ionic conductivity was elevated to 1.66 × 10^–2^ S cm^−1^ and t_+_ was significantly increased to 0.84. Li||Li symmetric cells maintained a stable overpotential of ~ 60 mV for 1500 h, revealing the PIEs can inhibit dendrite growth.

**Fig. 8:**
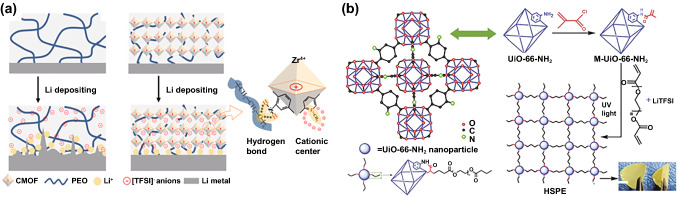
**a** Li plating behavior with PEO-LiTFSI and PEO-LiTFSI@CMOF [101]. Copyright 2019, Elsevier. **b** Synthesis process of the PEGDA-LiTFSI@UIO66-NH_2_ [148]. Copyright 2018, Royal Society of Chemistry

MOFs are comprised of inorganic clusters containing center metal ions and organic ligands [144]. In addition to sharing some characteristics with zeolites, including great thermal stability, large specific surface area and Lewis acidic surface, MOFs also contain their own distinct organic functional groups, which allow for the flexible control of surface properties [145, 146]. Unsaturated metal sites in MOFs can interact with anions to facilitate Li^+^ ion transport, hence enhancing ionic conductivity [147]. The periodic crystal structure and organized channels in MOFs provide uniform Li^+^ flux, ensuring uniform Li^+^ plating behavior and inhibiting dendrite growth. Stephan et al. enhanced the ionic conductivity of PEO-LiTFSI by two orders of magnitude with aluminum benzenetricarboxylate (Al-BTC) as filler [117]. The obtained PIEs exhibited excellent thermal stability and cycle stability to LMAs. They also reported that the insertion of aluminum terephthalate (Al-TPA) can reduce the migration of polysulfides in lithium–sulfur batteries and realize a stable cycle performance [118]. Zheng et al. constructed a 3D MOF network (Zirconium benzenedicarboxylate MOF, UIO-66) by electrospinning and then filled it with PAN/PEO-LiTFSI to obtain PIEs@UIO-66 (Fig. 7c) [100]. Density functional theory (DFT) demonstrated that UIO-66 had strong adsorption to Li^+^ ions. The interconnected particles offered continuous pathways for the rapid transport of Li^+^ ions, efficiently enhancing the ionic conductivity (2.89 × 10^–4^ S cm^−1^) and promoting the homogeneous distribution of Li^+^ flux. The PIEs@UIO-66 had high t_+_ (0.52), wide ESW (4.7 V), remarkable ability to suppress lithium dendrites and high mechanical strength. Guo et al. produced a novel cationic MOF (CMOF) by grafting pyridine onto UiO-66 and dispersed it in PEO-LiTFSI to form PIEs (Fig. 8a) [101]. CMOF fixed anions through electrostatic interaction and its large specific surface area further enhanced the adsorption of anions, making its t_+_ reach 0.72. Moreover, CMOF grafted with -NH_2_ groups protected the ether-oxygen on the polymer chains by hydrogen bonding, extending the electrochemical window to 4.97 V. After 300 cycles at 1C, the ASSBs combined with LMAs and LiFePO_4_ retained 85.4% of their initial capacity. Zhang et al. grafted polyethylene glycol diacrylate chain (PEGDA) onto vinyl-functionalized MOF nanoparticles (UIO66-NH_2_) through UV photopolymerization and formed PIEs with LiTFSI (Fig. 8b) [148]. The PIEs have a fivefold increase in ionic conductivity over PEGDA-LiTFSI, reaching 10^–5^ S cm^−1^.

#### Other Inert Fillers

Other inert inorganic materials, such as mixed metal oxides, phosphates, layered clay materials, are also widely used as fillers. Stephan et al. incorporated MgAl_2_O_4_ into PEO-LiPF_6_ to create PIEs by hot press [149]. The addition of MgAl_2_O_4_ improved the *T*_g_ and ionic conductivity of the polymer, which attributed to Lewis acid properties of MgAl_2_O_4_, can compete with Li^+^ ions and form complexes with PEO chains, thus decreasing polymer crystallization. Nanosized Ca_3_(PO_4_)_2_ was reported to produce a similar effect on the performance of PEO-LiTFSI and PEO-LiClO_4_ [150]. Nano-layered clays, such as montmorillonite and kaolinite, were utilized as inorganic fillers due to their high dielectric characteristics and specific surface area, which were conducive to the dissociation of lithium salts [151–153].

In summary, whereas inert fillers are unable to transport Li^+^ ions, numerous surface groups can interact with polymers and lithium salts to prevent polymer crystallization and promote lithium salt dissociation. Additionally, inert fillers can improve the mechanical and thermal stability of polymers. The interaction between inorganic fillers and anions can inhibit the continuous oxidative decomposition of anions and widen the ESW of the PIEs.

### Active Fillers

**Fig. 9:**
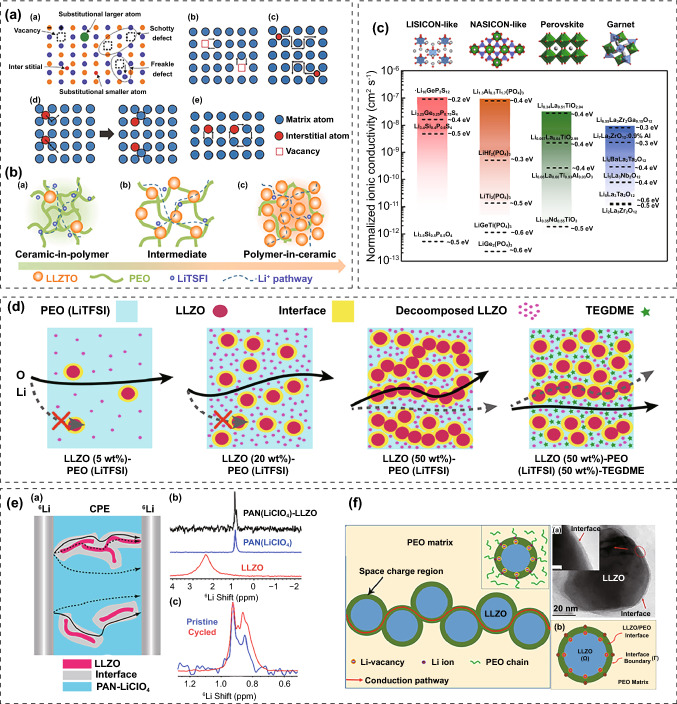
**a** Diffusion modes of Li^+^ ion in ISEs [28]. Copyright 2018, Royal Society of Chemistry. **b** Diffusion modes of Li^+^ ion in PIEs with active fillers [155]. Copyright 2018, Elsevier. **c** Crystal structure, conductivity and activation energy of different active fillers [156]. Copyright 2016, American Chemical Society. **d** Schematic diagram of Li^+^ ion diffusion routes in PEO-LiTFSI with different contents of LLZO fillers [157]. Copyright 2018, American Chemical Society. **e** Schematic of different Li^+^ diffusion pathways in the PIEs and ^6^Li NMR spectra of the PAN-LiClO_4_@LLZO, LLZO nanowires, PAN-LiClO_4_ and cycled PIEs [158]. Copyright 2017, American Chemical Society. **f** Schematic of the rapid ion diffusion route in space charge regions and the TEM image of space charge regions [159]. Copyright 2019, American Chemical Society

Active fillers allow efficient conduction of Li^+^ ions. Li^+^ ions exhibit different migration patterns in different regions of the PIEs with active fillers: (1) segment movement within the polymer, (2) vacancy or interstitial migration in the active fillers (Fig. 9a) and (3) interfacial migration between the fillers and polymer (Fig. 9b) [28, 154, 155]. Debates still exist regarding the migration paths of Li^+^ ions in PIEs containing active fillers, which will be described in depth in the following section. Based on the type of solid-state electrolyte used as fillers, they can be classified as garnet-type, NASICON-type, perovskite-type and sulfide-type PIEs (Fig. 9c) [156].

#### Garnet-Type PIEs

Thangadurai et al. reported the garnet-type Li_5_La_3_M_2_O_12_ (*M* = Nb, Ta) with an ionic conductivity of 10^–6^ S cm^−1^ at room temperature for the first time in 2003 [160]. By inserting more lithium atoms into the framework, a series of SSEs with garnet structure were created. Li_6.4_La_3_Zr_1.4_Ta_0.6_O_12_ has the highest bulk ionic conductivity of 10^–3^ S cm^−1^ at 25 °C among the known Li-rich garnets [161]. Garnet-type SSEs have the advantages of excellent ionic conductivity (~ 10^–4^–10^–3^ S cm^−1^), oxidation resistance under high voltage, stability to lithium metal and superior mechanical strength. Nevertheless, they also have the issue of significant interfacial resistance brought by inadequate contact with the rough interface [162]. Composited with polymer can accomplish robustness and flexibility, minimize interfacial contact impedance and overcome the poor processability of powder ceramics. The recent research on PIEs filled with garnet-type fillers and their properties is summarized in Table 3.

**Table 3:** PIEs filled with garnet-type fillers and their properties

Polymer matrix	Lithium salt	Fillers	Ionic conductivity (S cm^−1^)	ESW (V)	Filling ratio (wt%) and morphology	References
PAN	LiClO_4_	Li_7_La_3_Zr_2_O_12_	1.3 × 10^–4^ (30 °C)	5	5% nanowires	[158]
PEO	LiClO_4_	Li_6.75_La_3_Zr_1.75_Ta_0.25_O_12_	5 × 10^–4^ (25 °C)	–	10% microparticles	[163]
PEO	LiTFSI	Li_7_La_3_Zr_2_O_12_	2.4 × 10^–4^ (25 °C)	6	10% nanowires	[164]
PMMA	LiClO_4_	Li_6.75_La_3_Zr_1.75_Nb_0.25_O_12_	2.2 × 10^–5^ (25 °C)	5.5	10% nanowires	[165]
PVDF	LiClO_4_	Li_6.4_La_3_Zr_2_Al_0.2_O_12_	1.5 × 10^–4^ (30 °C)	4.7	20% nanoparticles	[166]
PEO	LiTFSI	Li_6.4_La_3_Zr_1.4_Ta_0.6_O_12_	2.1 × 10^–4^ (30 °C)	4.75	12.7 vol% nanoparticles	[87]
PEO	LiTFSI	Li_6.4_La_3_Zr_2_Al_0.2_O_12_	2.5 × 10^–4^ (30 °C)	6	7 mol% nanofibers	[167]
PVDF	LiClO_4_	Li_6.4_La_3_Zr_2_Al_0.2_O_12_	1.2 × 10^–4^ (30 °C)	6	75% nanofibers	[168]
PEO	LiClO_4_	Li_6.25_Al_0.25_La_3_Zr_2_O_12_	3.0 × 10^–4^ (24 °C)	5	70% nanoparticles	[169]
PEO	LiTFSI	Li_6.55_Ga_0.15_La_3_Zr_2_O_12_	4.5 × 10^–4^ (70 °C)	–	31% microparticles	[170]
PEO	LiTFSI	Li_6.75_La_3_Zr_1.75_Ta_0.25_O_12_	1.1 × 10^–5^ (25 °C)	5.5	40% microparticles	[171]
PPCL	LiTFSI	Li_6.75_La_3_Zr_1.75_Ta_0.25_O_12_	5.2 × 10^–4^ (20 °C)	4.6	5% nanoparticles	[172]
PEO	LiClO_4_	Li_7_La_3_Zr_2_O_12_	4.4 × 10^–4^ (50 °C)	6	52.5% microparticles	[173]
PEO	LiTFSI	Li_7_La_3_Zr_2_O_12_	0.9 × 10^–4^ (25 °C)	5.5	50% 3D frameworks	[174]
PEO-G4	LiTFSI	Li_7_La_3_Zr_2_O_12_	1 × 10^–4^ (20 °C)	> 4	40% microparticles	[175]

Gerbaldi et al. added Li_7_La_3_Zr_2_O_12_ (LLZO) fillers and a photoinitiator to the PEO-tetra (ethylene glycol dimethyl ether) (G4)-LiTFSI and then induced cross-linking under ultraviolet light to generate PIE films. The PIEs had good flexibility and exhibited an ionic conductivity more than 1 × 10^–4^ S cm^−1^ and a t_+_ greater than 0.5 at 20 °C. The Li||LiFePO_4_ cells with the PIEs demonstrated a remarkable specific capacity for 400 cycles [175]. The relationship between ion mobility, transport pathways and activity concentration in PEO-LiTFSI@LLZO was determined by solid-state nuclear magnetic resonance (NMR) [157]. The results demonstrated that when the LLZO content in the PIEs was less than 20 wt%, Li^+^ ions were mainly conducted through PEO (Fig. 9d). Once the concentration of LLZO reached a threshold level, the particles joined together, forming an infiltration network. Li^+^ ions migrated through the network rather than the PEO matrix. The critical concentration depended on several factors such as particle size, morphology as well as the dispersity of the fillers. The effects of LLZO fillers on ionic conductivity of PIEs are mainly manifested in the following aspects: (1) LLZO fillers reduced the crystallinity of polymer matrix; (2) Li^+^ ion channels in PEO could be blocked by LLZO particles and reduced the mobility of Li^+^ ions; (3) LLZO contributed as an extra source of Li^+^ ions to the conductivity. The trade-off between three competing effects determined whether the fillers increased or decreased ionic conductivity at a given concentration.

Chan et al. improved the ionic conductivity of PAN-LiClO_4_ by incorporating 5 wt% LLZO nanowires [158]. NMR revealed that LLZO nanowires changed the local environment in the polymer matrix and Li^+^ ion transport preferentially happened at the LLZO/polymer interface (Fig. 9e). The total ionic conductivity of PIEs adding LLZO nanoparticles (1.13 × 10^–5^ S cm^−1^) was much lower than that of PIEs adding LLZO nanowires (1.31 × 10^–4^ S cm^−1^). This indicated that the morphology and continuous conduction pathways provided by fillers were essential for the improvement of ionic conductivity.

**Fig. 10:**
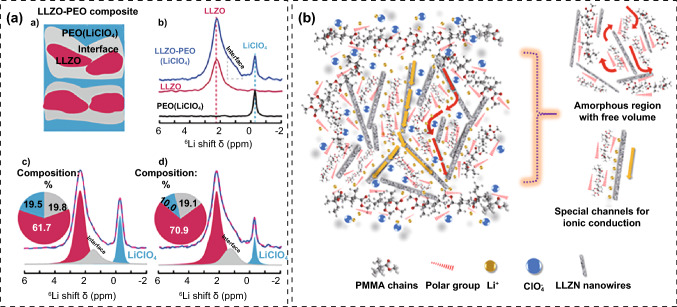
**a** NMR spectra and spectral simulation for LLZO, PEO-LiClO_4_ and PEO-LiClO_4_@LLZO [176]. Copyright 2019, John Wiley and Sons. **b** Multiple Li^+^ conduction forms in PMMA-LiClO_4_@LLZN [165]. Copyright 2019, Elsevier

Percolation effect may contribute significantly to the ionic conductivity of PIEs [157]. Wei et al. observed the space charge regions at the interface of PEO/Li_6.25_Ga_0.25_La_3_Zr_2_O_12_ (LLZO-Ga) nanoparticles by transmission electron microscope (TEM) [159]. Phase-field simulation demonstrated the chemical potential difference between LLZO-Ga and PEO drove the Li^+^ to migrate to the surface sites, leading to the enrichment of Li^+^ ions and low concentration of vacancies. As soon as the space charge region and phase distribution satisfied the criteria for establishing the percolation, percolation effect occurred, creating successive rapid transport routes and increasing ionic conductivity dramatically. Meanwhile, the space charge region surrounding isolated fillers barely impacted the ionic conductivity (Fig. 9f). Hu et al. tracked the Li^+^ diffusion paths in PEO-LiClO_4_@LLZO combining isotope labeling and Li NMR. By detecting that ^6^Li in the LMAs replaced ^7^Li in the PEO-LiClO_4_@LLZO, they found that Li^+^ ions diffused mainly through LLZO particles rather than through the interface or the polymer matrix (Fig. 10a) [176]. The aforementioned results imply that the observed Li^+^ diffusion path is closely related to the prepared PIEs inherently tied to the morphology, content, dispersion and properties of fillers.

Shen et al. suggested that Li_6.75_La_3_Zr_1.75_Ta_0.25_O_12_ (LLZTO) fillers can induce structure changes in PVDF [163]. La atoms of LLZTO can complex with N atoms and C=O groups of N, N-dimethylformamide (DMF) coupled with electron enrichment at the N atoms. The electron-rich N atoms acted as Lewis bases donated electron pairs and caused the partial dehydrofluorination of PVDF. The C=C on the modified PVDF enhanced the acid–base interaction with different components. LLZTO particles as Lewis acid promoted the dissociation of lithium salt and increased the concentration of Li^+^ ions. Partially dehydrofluorinated PVDF enhanced the interaction with LLZTO and further reduced the crystallinity of PVDF, resulting in enhanced comprehensive performance of the PIEs.

Wang et al. suggested that Li_6.75_La_3_Zr_1.75_Nb_0.25_O_12_ (LLZN) nanowires can interact with C=O and O=C–N, which were left by the solvent (DMF) [165]. The interaction reinforced the connection between fillers and polymer thus creating abundant amorphous regions and large free volume for segment movement. The surface group of ceramic filler had strong adsorption for ClO_4_^−^, hence facilitating the dissociation of the salt. Moreover, the vacancies of the LLZN nanowires provided special conductive channels for ion transportation. The multiple Li^+^ conduction forms significantly increased the ionic conductivity of PIEs (Fig. 10b).

#### NASICON-Type PIEs

The primary NASICON-type SSEs are derived from LiGe_2_(PO_4_)_3_ and LiTi_2_(PO_4_)_3_. The ionic conductivity can be further enhanced by partial replacement of tetravalent Ge^4+^ and Ti^4+^ with trivalent cations such as Ga^3+^, Al^3+^ and Fe^3+^. The ionic conductivity of Li_1.3_Al_0.3_Ti_1.7_(PO_4_)_3_ (LATP) can reach 10^–3^ S cm^−1^ and satisfy the requirements of SSEs [177]. Moreover, they are resistant to air and water, enabling large-scale synthesis and battery assembly in an air atmosphere, which decreases processing challenge and cost [178]. While they have an issue with instability to lithium since Ti^4+^ and Ge^4+^ are easily reduced, generating high-impedance interfacial phases [179, 180]. Composited with polymer electrolyte owning electronic insulation and flexibility can improve electrochemical and contact stability on the interface of NASICON-type SSEs. The recent research on PIEs filled with NASICON-type ISEs and their properties is summarized in Table 4.

**Table 4:** PIEs filled with NASICON-type fillers and their properties

Polymer matrix	Lithium salt	Fillers	Ionic conductivity (S cm^−1^)	ESW (V)	Filling ratio (wt%) and morphology	References
PEO/PEG	LiTFSI	Li_1.5_Al_0.5_Ge_1.5_(PO_4_)_3_	1.67 × 10^–4^ (25 °C)	–	26% nanoparticles	[181]
PEO	LiTFSI	Li_1.5_Al_0.5_Ge_1.5_(PO_4_)_3_	1.25 × 10^–4^ (25 °C)	> 3.8	90.8% brick-and-mortar	[182]
PVDF	LiTFSI	Si@LiTi_2_(PO_4_)_3_	1.06 × 10^–3^ (25 °C)	4.86	30% nanoparticles	[183]
PEO	LiTFSI	Li_1.3_Al_0.3_Ti_1.7_(PO_4_)_3_	7.47 × 10^–4^ (60 °C)	5.1	62.7% 3D framework	[184]
PVDF-HFP	LiTFSI	Li_1+*x*_Al_x_Ge_2−*x*_ (PO_4_)_3_	0.96 × 10^–3^ (25 °C)	4.8	50% nanoparticles	[185]
PEO	LiTFSI	Li_1.4_Al_0.4_Ge_1.6_(PO_4_)_3_	1.72 × 10^–4^ (25 °C)	–	Submicroparticles	[186]
PEO	LiTFSI	Li_1.5_Al_0.5_Ge_1.5_(PO_4_)_3_	1.67 × 10^–4^ (20 °C)	5	60% microparticles	[187]
PEO	LiTFSI	Li_1.5_Al_0.5_Ge_1.5_(PO_4_)_3_	4.4 × 10^–5^ (25 °C)	5.1	20% microparticles	[188]
PEO	LiTFSI	Li_1.5_Al_0.5_Ge_1.5_(PO_4_)_3_	0.9 × 10^–4^ (30 °C)	5.12	99% microparticles	[189]
PPO	LiTFSI	Li_1.5_Al_0.5_Ge_1.5_(PO_4_)_3_	3.46 × 10^–4^ (25 °C)	4.78	75% microparticles	[190]
PPC	LiTFSI	Li_1.5_Al_0.5_Ge_1.5_(PO_4_)_3_	1.55 × 10^–4^ (25 °C)	> 4	70% nanoparticles	[191]
PEO	LiClO_4_	Li_1+*x*_Al_x_Ti_2−*x*_ (PO_4_)_3_	0.52 × 10^–4^ (25 °C)	4.8	40 vol% vertically aligned	[192]
PVDF-HFP	LiTFSI	Li_1.5_Al_0.5_Ti_1.5_(PO_4_)_3_	2.3 × 10^–4^ (25 °C)	> 4	30% microparticles	[193]
PEO	LiTFSI	Li_1.3_Al_0.3_Ti_1.7_(PO_4_)_3_	4.0 × 10^–5^ (25 °C)	–	70% nanoparticles	[194]
PVDF-PMMA	LiTFSI	Li_1.3_Al_0.3_Ti_1.7_(PO_4_)_3_	1.23 × 10^–3^ (25 °C)	4.8	60% submicroparticles	[195]

**Fig. 11:**
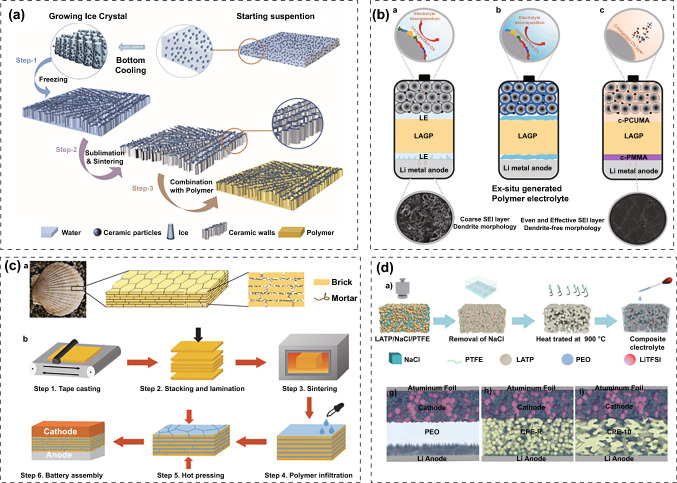
**a** Schematic of constructing a vertically aligned PIEs with LAGP and flexible PEO/PEG polymer [181]. Copyright 2019, Elsevier. **b** Comparison of interfacial evolution in a liquid electrolyte, ex situ polymer modification and in situ Janus polymer modification [196]. Copyright 2022, Elsevier. **c** Preparation process of PEO-LiTFSI@LAGP PIEs with “brick–mortar” microstructures [182]. Copyright 2020, John Wiley and Sons. **d** Illustration for the preparation of porous interconnected LATP networks with NaCl as a sacrificial template and SEM images of the PIEs [184]. Copyright 2019, Elsevier

Rational structural design can help ceramic and polymer electrolytes overcome their drawbacks and exploit their full potential. Yang et al. constructed PIEs with vertically aligned Li_1.5_Al_0.5_Ge_1.5_(PO_4_)_3_ (LAGP) and flexible PEO/PEG polymer (Fig. 11a) [181]. The vertical arranged LAGP created successive pathways for rapid ion diffusion and the PEO/PEG matrix made the PIEs flexible. The ionic conductivity of the PIEs reached 1.67 × 10^–4^ S cm^−1^ at 25 °C. After 300 cycles, the ASSBs built with LiFePO_4_ and LMAs retained 93.3% of the initial capacity. Jiang et al. adopt Janus interface modification strategy to improve the electrochemical stability at LAGP/electrodes interface. They sandwiched LAGP disks between in situ cross-linked PMMA and poly(cyclic carbonate urethane methacrylate)-based polymer electrolytes (Fig. 11b). Polymer electrolyte coatings not only kept PIEs in contact with the electrode, accelerating the interfacial ion transport kinetics, but also built stable CEI and SEI layers. The PIEs enabled the Li||LiNi_0.8_Mn_0.1_Co_0.1_O_2_ cells to have outstanding cycle stability at 4.5 V [196]. Yang et al. developed PIEs with “brick–mortar” microstructures (Fig. 11c) [182]. They prepared multilayer PEO-LiTFSI@LAGP by stacking and sintering at 850 °C. Then, the stack was immersed in polymer electrolyte under vacuum, and compressed at 80 °C to break into thin sheet and enable polymer to plug all gaps. The obtained PIEs exhibited extremely high ultimate bending strength and remarkable toughness. The ASSBs assembled with LiFePO_4_ and LMAs can retain 92% of their initial capacity at 0.5C after 300 cycles at 60 °C. Xiong et al. embedded silane functionalized LATP nanoparticles into the PVDF framework by electrospinning to form nanofiber membranes and then carried out thermal initiation polymerization of vinylene carbonate-based precursors in the composite network (Si@LATP/PVDF/PVC) [183]. Silane functionalization increased the affinity of Si@LATP with the PVDF skeleton and fully exposed the Lewis acid sites on LATP. The -NH_3_^+^ in poly-siloxane further increased the anion adsorption. The PIEs possessed high electrochemical stability to lithium and the ASSBs coupled with LiNi_0.5_Co_0.2_Mn_0.3_O_2_ exhibited excellent cycle performance and rate capability. Fan et al. created porous interconnected LATP networks with NaCl as a sacrificial template and introduced PEO-LiTFSI into the networks (Fig. 11d). The PIEs not only served as rapid transport routes for Li^+^ ions, but also as physical barriers to prevent the growth of Li dendrites [184].

#### Perovskite-Type PIEs

Perovskite-type SSEs include Li_3x_La_2/3−*x*_TiO_3_ and (Li, Sr)(*M*, *M*') O_3_ (*M* = Ti, Hf, Zr, Ga, Sn, etc., *M*' = Ta, Nb, etc.) [197, 198]. They process high ionic conductivity at room temperature (10^–3^ S cm^−1^) as well as outstanding mechanical strength and electrochemical oxidation potential (> 8 V). But they are vulnerable to reduction by the LMAs (Ti^4+^  + Li → Ti^3+^  + Li^+^). It is effective in overcoming defects by compositing with polymers. The recent research on PIEs filled with perovskite-type ISEs and their properties is summarized in Table 5.

**Table 5:** PIEs filled with perovskite-type fillers and their properties

Polymer matrix	Lithium salt	Fillers	Ionic conductivity (S cm^−1^)	ESW (V)	Filling ratio (wt%) and morphology	References
PEO	LiTFSI	LSTZ	5.4 × 10^–5^ (25 °C)	5.2	20% 1 μm particles	[199]
PEO	LiTFSI	Li_0.35_La_0.55_TiO_3_	8.8 × 10^–5^ (25 °C)	5.1	44% 3D frameworks	[200]
PEO	LiTFSI	Li_0.33_La_0.557_TiO_3_	0.13 × 10^–3^ (25 °C)	> 3.8	Vertically aligned nanoparticles	[201]
PAN	LiClO_4_	Li_0.33_La_0.557_TiO_3_	2.4 × 10^–4^ (25 °C)	4	15% nanowires	[202]
PAN	LiClO_4_	Li_0.33_La_0.557_TiO_3_	6.05 × 10^–5^ (25 °C)		3% well-aligned nanowires	[203]
PEO	LiTFSI	Li_0.33_La_0.557_TiO_3_	0.16 × 10^–3^ (25 °C)	4.7	23% nanofiber	[204]
PEO/PPC	LiTFSI	Li_0.33_La_0.557_TiO_3_	5.7 × 10^–5^ (30 °C)	6	8% nanowires	[205]
PEO	LiTFSI	Li_0.33_La_0.557_TiO_3_	2.4 × 10^–4^ (25 °C)	5	15% nanofibers	[206]
PEO	LiTFSI	Li_0.3_La_0.557_TiO_3_	1.8 × 10^–4^ (25 °C)	4.5	20% nanofibers	[207]
PEO	LiTFSI	Li_0.3_La_0.557_TiO_3_	2.3 × 10^–4^ (25 °C)	–	23.7% nanofibers	[208]

**Fig. 12:**
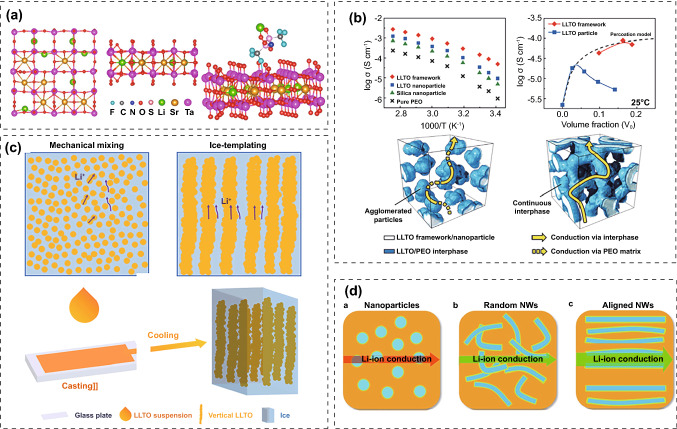
**a** Structure of the LSTZ and LiTFSI adsorbed on the surface of the LSTZ [199]. Copyright 2019, Proceedings of the National Academy of Sciences.** b** Ionic conductivity and percolation model of LLTO networks and LLTO nanoparticles [200]. Copyright 2018, John Wiley and Sons. **c** Schematic diagram of Li^+^ ion conduction in continuous network and randomly scattered LLTO [201]. Copyright 2020, Elsevier. **d** Schematic diagram of Li^+^ ion conduction in randomly scattered and well-aligned nanowires [203]. Copyright 2017, Springer Nature

Hu et al. reported flexible PIEs made of PEO-LiFSI and Li_3/8_Sr_7/16_Ta_3/4_Zr_1/4_O_3_ (PEO-LiFSI@LSTZ). The increased bonding of Ta^5+^ to F atoms in anions accelerated the release of Li^+^ ions and improved ionic conductivity (Fig. 12a) [199]. Concurrently, the SEI layer formed on LMAs increased the interfacial stability and inhibited lithium dendrites. The symmetrical Li||Li cells with PEO-LiFSI@LSTZ exhibited long-life stripping/plating behavior over 700 h. ASSBs matched with LiFePO_4_ or LiNi_0.8_Co_0.1_Mn_0.1_O_2_ exhibited high cycle stability and rate performance.

The alignment of the fillers in the polymer matrix has a significant effect on ionic conductivity and cell performance. Yu et al. compared randomly distributed Li_0.35_La_0.55_TiO_3_ (LLTO) fillers with 3D interconnection network on the performance of PIEs (Fig. 12b) [200]. In the random distribution structure, the agglomeration of nanoparticles generated discontinuous Li^+^ conductive paths which reduced the percolation behavior and ionic conductivity. LLTO with a 3D interconnection structure provided a continuous interface phase for Li^+^ conduction, which can significantly improve the ionic conductivity of PIEs. Zhao et al. fabricated PIEs with a vertically aligned LLTO framework embedded in a PEO-LiTFSI matrix [201]. The vertically aligned structure provided a rapid and continuous network for Li^+^ transport, obtaining ionic conductivity of 0.13 × 10^–3^ S cm^−1^, which was 2.4 times more than that of PIEs with randomly scattered LLTO (Fig. 12c). Cui et al. investigated the impact of Li_0.33_La_0.557_TiO_3_ nanowire orientation on the ionic conductivity of PIEs. Compared to the nanowires that were randomly scattered, well-aligned nanowires had a ten-fold increase in ionic conductivity (6.05 × 10^–5^ S cm^−1^ at 30 °C) [203] (Fig. 12d). Cui et al. compared the effect of Li_0.33_La_0.557_TiO_3_ nanoparticles with nanowires on the performance of PAN-LiClO_4_. Nanowires allowed for continuous ion transport channels, which shortened the transport distance and increased ionic conductivity compared to nanoparticle packing, where Li^+^ ions must cross several particle–particle junctions [202]. Therefore, developing continuous conduction paths is crucial for achieving high ionic conductivity of PIEs.

#### Sulfide-Type PIEs

**Fig. 13:**
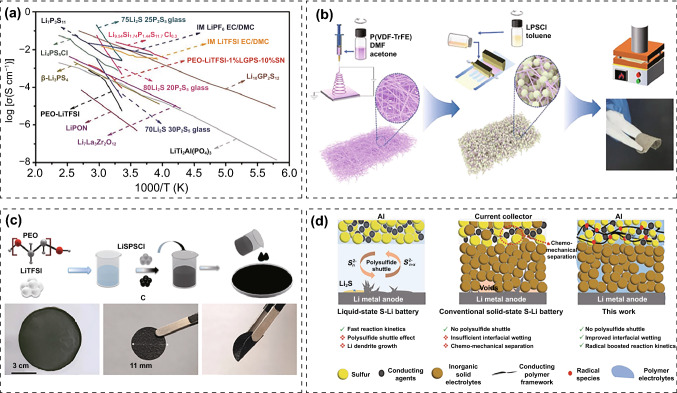
**a** Arrhenius of sulfide electrolytes compared to organic liquid electrolytes [209]. Copyright 2019, John Wiley and Sons. **b** Preparation process of PIEs with Li_6_PS_5_Cl (LPSCl) and P(VDF-TrFE) by electrospinning-permeation-hot pressing method [88]. Copyright 2022, John Wiley and Sons. **c** Preparation process of PEO-LiTFSI@LSPSCl [211]. Copyright 2022, John Wiley and Sons. **d** Comparison of S||Li batteries operated in liquid electrolytes, ceramic solid-state electrolytes and PIEs [212]. Copyright 2020, John Wiley and Sons

The ionic conductivity of sulfide-type SSEs can reach ~ 10^–2^ S cm^−1^, while the electrochemical stability and interfacial stability are poor (Fig. 13a) [209]. The sensitivity of sulfides to air necessitates treatment in inert gas environment, which impedes their large-scale utilization. Sulfide-type SSEs are classified as binary or ternary based on their compositions. Binary sulfide SSEs comprise P_2_S_5_ and Li_2_S including Li_7_P_3_S_11_ and Li_3_PS_4_, while ternary sulfide electrolytes comprise P_2_S_5_, Li_2_S and MS_2_ (*M* = Si, Ge, Sn) including Li_10_GeP_2_S_12_ (LGPS) and Li_6_PS_5_*X* (*X* = Cl, Br, I) [210]. Combining sulfide-type SSEs with polymers can increase interfacial stability and improve processability. The recent research on PIEs filled with sulfide-type ISEs and their properties is summarized in Table 6.

**Table 6:** PIEs filled with sulfide-type fillers and their properties

Polymer matrix	Lithium salt	fillers	Ionic conductivity (S cm^−1^)	ESW (V)	Filling ratio (wt.%) and morphology	References
PEO	LiTFSI	Li_10_GeP_2_S_12_	1.8 × 10^–4^ (25 °C)	> 3	2% microparticles	[212]
PVDF	LiTFSI	78Li_2_S-22P_2_S_5_	5.3 × 10^–4^ (25 °C)	> 3	97% microparticles	[213]
PGMA	–	Li_3_PS_4_	1.8 × 10^–4^ (25 °C)	4.8	50% microparticles	[214]
PVDF-HFP	LiTFSI	Li_7_PS_6_	1.1 × 10^–4^(25 °C)	> 4	10% microparticles	[215]
PEO-CTMS	LiTFSI	Li_10_GeP_2_S_12_	2.4 × 10^–4^ (25 °C)	4.7	77.5% microparticles	[216]
PEO	LiTFSI	Li_10_GeP_2_S_12_	2.2 × 10^–4^ (25 °C)	–	70% microparticles	[217]
PEO	LiTFSI	Li_6_PS_5_Cl	3.6 × 10^–3^ (80 °C)	–	40% microparticles	[218]
PEO	LiClO_4_	Li_6_PS_5_Cl + SiO_2_	3 × 10^–3^ (25 °C)	> 4.2	95% microparticles	[219]
PVDF	LiTFSI	3Li_2_S·2P_2_S_5_	3.4 × 10^–4^ (25 °C)	> 3.8	33% nanoparticles	[220]
PEO	LiTFSI	Li_6_PS_5_Cl	1.1 × 10^–3^ (25 °C)	4.9	97% microparticles	[221]
PEO	LiTFSI	Li_10_SnP_2_S_12_	1.7 × 10^–4^ (50 °C)	5	1% microparticles	[222]
P(VDF-TrFE)	–	Li_6_PS_5_Cl	1.3 × 10^–3^ (25 °C)	5	79% microparticles	[88]
PEO	–	Li_6_PS_5_Cl	1.0 × 10^–3^ (80 °C)	> 4	95% microparticles	[223]

Nan et al. prepared ultra-thin and flexible PIEs with Li_6_PS_5_Cl (LPSCl) and poly(vinylidene fluoride-co-trifluoroethylene) (P(VDF-TrFE)) by electrospinning-permeation-hot pressing method (Fig. 13b) [88]. The TrFE groups allowed P(VDF-TrFE) to exhibit dominant-phase with an all-trans conformation, resulting in a higher dielectric constant and greater flexibility than PVDF. The strong polarity of the polymer promoted the interaction with LSPSCl. The P(VDF-TrFE) network enabled the complete infiltration of LPSCl particles to generate interpenetrating P(VDF-TrFE)@LPSCl films. The PIEs had an ionic conductivity of up to 1.2 × 10^–3^ S cm^−1^ and enabled Li-In||LiNi_0.8_Co_0.1_Mn_0.1_O_2_ cells to maintain 71% capacity after 20,000 cycles at 1.0 mA cm^−2^. PEO-LiTFSI@LSPSCl PIEs were developed by solution casting method (Fig. 13c) [211]. The Li||S batteries assembled by the PIEs retained 97.8% of their initial capacity at 0.1 Ag^−1^. Cryo-TEM revealed that LSPSCl facilitated the decomposition of TFSI^−^ and enhanced ionic conductivity. Li_2_O, LiF and Li_2_S-rich SEI formed by anionic decomposition hindered dendrite growth and enhanced interfacial stability. PEO-LiTFSI@LSPSCl also suppressed the shuttling of phosphorus and sulfur specie. By employing PEO-LiTFSI@Li_3.25_Ge_0.25_P_0.75_S_4_, Bieker et al. reduced the interfacial contact impedance and increased ionic conductivity (0.42 × 10^–3^ S cm^−1^) and t_+_ (0.87). The cells made of vulcanized polyacrylonitrile and LMAs exhibited outstanding rate performance and cycle stability (Fig. 13d) [212].

### Synthesis of PIEs

The preparation methods of PIEs are mainly based on the synthesis of polymers involved solution casting, phase inversion, electrospinning and in situ polymerization. Solution casting entails dispersing the polymer, lithium salts and fillers in solvents, thoroughly agitating and then casting the mixture onto a flat substrate [224]. After removing the solvents, PIEs are obtained. This procedure is straightforward to implement; however, it cannot precisely regulate the porosity and thickness of PIEs. Phase inversion and solution casting share similar beginning steps; however in the former, the mixture coated on the substrate is soaked in a nonsolvent to replace the solvent. The exchange process causes phase transitions in the polymer. After drying at a high temperature, porous PIEs are created. Electrospinning is commonly used to fabricate one-dimensional nanomaterials and nanofiber-woven 3D networks. It can produce PIEs with adjustable porosity, pore size, thickness and excellent elasticity. Long fibers can offer continuous routes for ion transport [16]. In situ polymerization is the process of solidifying procurers containing curable monomers (e.g., tetrahydrofuran, 1,3-dioxolane, etc.), initiators (e.g., PF_5_, BF_3_, AlCl_3_, etc.), lithium salts and inorganic fillers under specific conditions (e.g., heat, UV radiation) [225]. Inorganic fillers shall be uniformly dispersed in the polymer during the process. Grafting allows fillers to covalently join on the polymer to avoid agglomeration of nanoparticles [226].

When solvent treatment is performed, the compatibility between solvents and fillers must be evaluated. In the presence of sulfides, the polarity index of the solvent must be less than 3.1 [227]. Physical and chemical properties of different PIE components, such as reactivity and toxicity with wet air and oxygen, must be thoroughly accounted for. For example, sulfides exhibit strong reactivity in humid air, leading to the creation of hazardous H_2_S [228]. PIEs composed of sulfides must be treated in a dry environment or even an inert gas atmosphere [229].

Generally, active fillers can conduct Li^+^ ions and the interface generated by their contact with polymers can provide transport routes for Li^+^ ions. Establishing continuous conduction routes is critical to improving ionic conductivity. The fillers’ type, particle size, shape, arrangement and interaction with other components will influence performance. Vertical heterostructures possess asymmetrical features, which can enrich the design strategies and show great potential in the practical application of ASSBs.

## Summary and Perspective

**Fig. 14:**
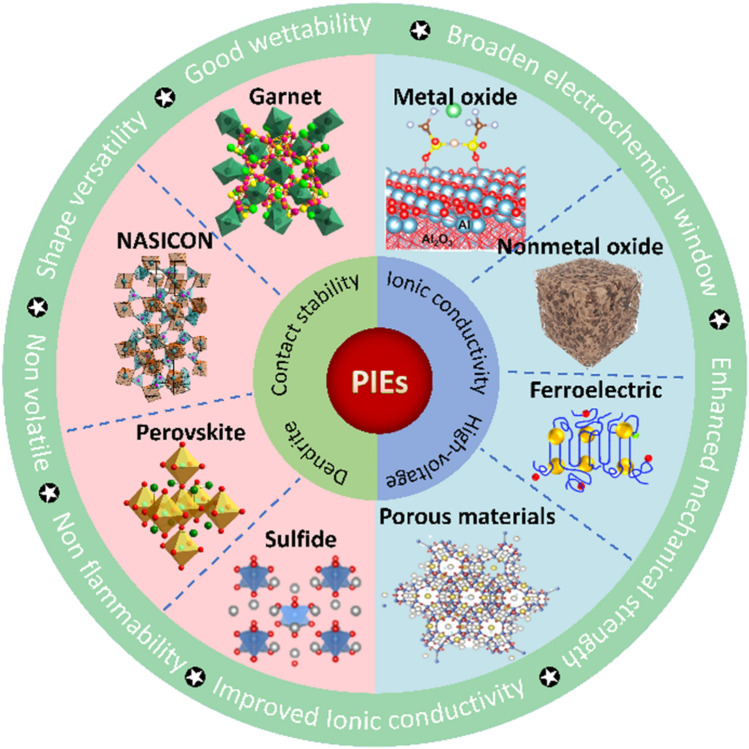
Overview of the topics in this paper

This review presents recent progress on PIEs with inorganic fillers and focuses on the influence of inert and active fillers on the characteristics of the PIEs (Fig. 14). Especially, composite with active fillers can effectively overcome defects of the single component and improve the comprehensive performance of the electrolyte. The characteristics of PIEs are influenced by the type, content, morphology, arrangement and surface groups of the fillers. Proper design of fillers can significantly improve the ionic conductivity, mechanical strength and interfacial stability of the PIEs. Given their superior integrative performance, PIEs have been extensively investigated in ASSBs assembled with high-energy-density cathode and anode including S, O_2_ and LMAs. Even though PIEs have made significant strides, fundamental scientific questions remain and widespread implementation confronts substantial obstacles.

1. Even though ionic conductivity of PIEs has greatly increased compared to traditional PSEs, it is still much lower than that of conventional liquid electrolytes, which is detrimental to develop LIBs with high energy density and power capability. Precisely regulating the characteristics and arrangement of fillers are expected to break through the ionic conductivity limit of PIEs. Understanding the Li^+^ migration routes and interactions between different components can provide crucial theoretical direction for enhancing ionic conductivity. Furthermore, it is essential to develop advanced in situ characterization techniques and theoretical computation methods to conduct mechanistic investigations in PIEs.
2. It is critical for realizing the interfacial stability between PIEs and electrodes. Although the flexible polymer matrix can improve the contact with the rough electrode, the frequent expansion and contraction of the electrodes would degrade the contact during the charging/discharging processes. Especially when matching LMAs, the uneven plating/stripping behavior may cause the loss of electrical connection of active lithium. Regulating the electrochemical behavior of LMAs and adding minuscule ionic liquid or liquid electrolyte can considerably optimize surface contact.
3. Increasing the operating voltage is a potential avenue for developing high-energy–density batteries. By crafting the arrangement of the fillers and polymer, we can increase the stability on the high-voltage cathode and reduce dendritic growth and side reactions on the anode. Coatings that can withstand high voltages and reduction are expected to encourage the widespread use of PIEs.
4. At present, the thickness of PIEs is still significantly higher than that of commercial polyolefin separators. Developing ultra-thin PIEs with moderate rigidity and flexibility is conducive to improving the energy density of batteries. The uniform dispersion of inorganic fillers in polymer matrix facilitates to construct continuous and uniform Li^+^ transport channels. And the efficient dispersion of fillers is critical in the manufacture of PIEs. Meanwhile, processing compatibility with electrodes or other internal components must be guaranteed throughout synthesis and operation.

Generally, solving these problems still requires the joint efforts of multidisciplinary fields. The assessment of advanced research and outlook for future research in this paper is expected to benefit the next generation of all-solid-state lithium metal batteries.
